# Gut microbiota in vaccine naïve Gabonese children with rotavirus A gastroenteritis

**DOI:** 10.1016/j.heliyon.2024.e28727

**Published:** 2024-03-30

**Authors:** Gédéon Prince Manouana, Salih Kuk, Le Thi Kieu Linh, Srinivas Reddy Pallerla, Sandra Niendorf, Peter G. Kremsner, Ayola Akim Adegnika, Thirumalaisamy P. Velavan

**Affiliations:** a Centre de Recherches Médicales de Lambaréné, Lambaréné, Gabon; b Institute of Tropical Medicine, Universitätsklinikum Tübingen, Tübingen, Germany; c Vietnamese-German Center for Medical Research (VG-CARE), 10000, Hanoi, Viet Nam; d Department of Infectious Diseases, Robert Koch Institute, Berlin, Germany; e German Center for Infection Research (DZIF), Tübingen, Germany; f Fondation pour la Recherche Scientifique, Cotonou, Benin; g Faculty of Medicine, Duy Tan University, 50000, Da Nang, Viet Nam

**Keywords:** Rotavirus A, Diarrhoea, Children, 16S rRNA, Gut microbiota, Gabon

## Abstract

**Background:**

While the gut microbiome modulates the pathogenesis of enteric viruses, how infections caused by rotavirus A (RVA), with or without diarrhoea, alter the gut microbiota has been sparsely studied.

**Methods:**

From a cohort of 224 vaccine naïve Gabonese children with and without diarrhoea (n = 177 and n = 67, respectively), 48 stool samples were analysed: (i) RVA with diarrhoea (n = 12); (ii) RVA without diarrhoea (n = 12); (iii) diarrhoea without RVA (n = 12); (iv) healthy controls without diarrhoea and RVA (n = 12). The 16S rRNA metabarcoding using Oxford Nanopore sequencing data was analysed for taxonomic composition, abundance, alpha and beta diversity, and metabolic pathways.

**Findings:**

Alpha diversity showed that children with acute diarrhoea (with and without RVA infection), and children with acute diarrhoea without RVA had low microbial diversity compared to healthy children (p = 0.001 and p = 0.006, respectively). No significant differences observed when comparing children with RVA with or without diarrhoea. Beta diversity revealed high microbial heterogeneity in children without diarrhoea. Proteobacteria (68%) and Firmicutes (69%) were most common in the diarrhoea and non-diarrhoea groups, respectively. Proteobacteria (53%) were most common in children without RVA, while Firmicutes (55%) were most common with RVA. At the genus level, *Escherichia* (21%), *Klebsiella* (10%) and *Salmonella* (4%) were abundant in children with diarrhoea, while *Blautia* (11%), *Clostridium* (8%), *Lachnoclostridium* (6%) and *Ruminococcus* (5%) were abundant in children without diarrhoea. Metabolites involved in amino acid, carbohydrate, lipid, nucleotide, and vitamin metabolism were quantitatively altered.

**Interpretation:**

Although host physiology dictates the intestinal milieu, diarrhoea per se can alter a balanced gut microbiota, whereas infectious diarrhoea disrupts the gut microbiome and reduces its diversity.

## 1 Research in context (248 words)

### 1.1 Evidence before this study

The abundance and composition of the gut microbiota plays an important role in immune homeostasis and can be altered by enteric infections such as the one caused by rotavirus A (RVA). We searched PubMed for publications up to 2021 using the search terms “rotavirus A″ AND “gut microbiota” AND “children”. We have found only one report investigating the effects of the RotaTeq® vaccine against rotavirus A on the gut microbiota. However, there are no studies that have examined the gut microbiome in RVA-related enteric infections, particularly in rotavirus vaccine-naïve children under five years of age.

### 1.2 Added value of this study

There is limited data on the effects of RVA infection and diarrhoea on the composition and diversity of the gut microbiome. To our knowledge, this is the first study to investigate the diversity of the gut microbiota in children with diarrhoea and/or RVA infection. We observe that diarrhoea per se affects the diversity and richness of the gut microbiota. More importantly, there is a clear association between RVA and the relative richness of the gut flora in unvaccinated Gabonese children.

### 1.3 Implications of all the available evidence

Our results show that diarrhoea and RVA infection per se are two independent variables that can modulate gut microbial composition. This has important implications for our mechanistic understanding of host-microbiota interactions and for the development of an effective treatment strategy for diarrhoeal disease with or without RVA, where large parts of sub-Saharan Africa have not adopted rotavirus vaccination.

## 2 Introduction

Diarrhoea is a global health problem, especially among children. Childhood diarrhoea accounts for approximately 63% of the global diarrhoea burden [1,2], and is the second significant cause of infant mortality in developing countries [3,4]. Rotavirus A (RVA) is a common gastrointestinal pathogen in infants and children <5 years of age [5]. Despite the global introduction of vaccines against human rotavirus infection more than a decade ago, RVA still causes more than 200,000 deaths per year [6].

The gut microbiota plays an important role in hosts health. It participates in digestion and nutrient absorption and plays an essential role in priming gut-associated lymphoid tissue (GALT) that helps inhibit local or systemic infections [7]. The ecology and function of the microbiota have been shown associated with enteric viral infections [8]. In addition, viral infections can alter both the microbiota composition and the activity of the gut microbiome. For instance, poliovirus, norovirus and murine RVA have been reported to affect the gut microbiota [9]. In addition, commensal bacteria have been shown to enhance the infectivity of enteric viruses through several mechanisms, such as bacterial stabilization of viral particles, assisting target cells in viral adsorption and limiting antiviral immune responses [10]. RVA infection has been shown to reduce the diversity of the microbiota compared to norovirus infection, indicating a pathogen-specific interaction [11]. RVA infection temporally alters the gut microbiota by decreasing bacterial diversity and increasing the occurrence of opportunistic pathogens, such as *Shigella* sp.[12].

The gut microbiome has been implicated in the pathogenesis of several viruses, including adenovirus, astrovirus, calicivirus, coronaviruses, norovirus, poliovirus and rotavirus [8,10]. In addition, studies have shown that the gut microbiome can modulate immune responses to the rotavirus vaccine (RVV) [13,14]. It has been reported that the efficacy and effectiveness of RVV vaccination in children in low-income countries was significantly lower than in high-income countries, and the gut microbiota is thought to regulate RVV responses [13,15]. Certain bacterial taxa known to modulate infection course and immune responses, and identification of specific taxon mediating effects on RVA may provide mechanistic insights. In addition, pre-existing disorders of the gut microbiota make children more susceptible to enteropathogens colonisation and diarrhoeal disease.

The literature on the implication of the gut microbiome in the pathogenesis of RVA infections is still limited and further studies are necessary. As a first step, we aimed to understand the variations in gut microbiota in children under 5 years of age suffering from diarrhoea with or without RVA infection.

## 3 Methods

### 3.1 Ethics statement

The study protocol was approved by the Institutional Ethical Committee of the Centre de Recherches Médicales de Lambaréné (CERMEL) (CEI-CERMEL: 003/2017). Parents or legal representatives of the children signed the written informed consent before enrolment.

### 3.2 Study population

**Fig. 1:**
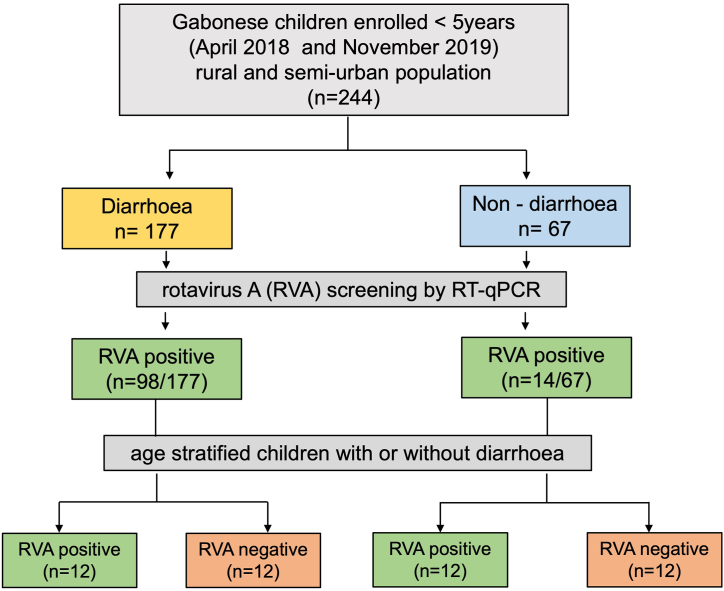
Flow chart on investigated study population [17]; RVA -rotavirus A; RT-qPCR - Quantitative reverse transcription polymerase chain reaction.

Children residing in Lambaréné, Gabon and the surrounding rural region were recruited for this study. The study included randomly selected 48 stool samples collected between April 2018 and November 2019 from a well-characterised cohort of 224 hospitalised children <5 years of age with diarrhoea (n = 177) and non-diarrhoea (n = 67) community controls [16] (Fig. 1). RVA was diagnosed in 112 of them (98/177 with diarrhoea and 14/67 without diarrhoea) [16,17].

### 3.3 Stool DNA extraction

DNA were extracted from 0.25g of faecal sample using QIAamp PowerFecal DNA kit (Qiagen GmbH, Hilden, Germany), according to the manufacturer instructions. Samples were mechanically disrupted using bead-beating for 6.5 m/s for 45 s with FastPrep-24™ 5G Instrument (MP Biomedicals™, Germany). DNA quality and quantity was assessed with the Qubit™ 4 fluorometer using the Qubit™ dsDNA BR Assay Kit (Thermo Fisher Scientific, Waltham, MA, USA).

### 3.4 16S rRNA sequencing with Oxford Nanopore MinION

For multiplex sequencing, the 16S rRNA was amplified from the faecal DNA using 16S barcoding kit SQK-16S024 (Oxford Nanopore Technologies, Oxford, UK) following the manufacturer's instructions. The ZYMOBIOMICS™ Microbial community DNA Standard was used as reference sample. Post-PCR, the amplicons were purified using AMPure XP beads, quantified using Qubit™ 4 fluorometer using the Qubit™ dsDNA BR Assay Kit (Thermo Fisher Scientific, Waltham, MA, USA). The amplicons were pooled with a total of 80–90 ng in 10 μL (6ng/sample). Sequencing was performed on a R9.4.1 flow cell on MinIONTM for 48 h (Oxford Nanopore Technologies, Oxford UK) using MinKNOW software (v.18.01.6). Subsequently, following Nanopore sequencing, high accuracy base calling of raw fast5 reads were base called to Fastq reads using Guppy (v3.4.3). The Fastq data was deposited in the European Nucleotide Archives (ENA) and are available under accession numbers ERR11758216-ERR11758263.

### 3.5 Data analysis

For data analysis, >100,000 fastq reads were used. The reads were quality filtered and the centrifuge pipeline was used to generate Operational Taxonomic Units (OTUs). Taxonomic designations were assigned by using the NCBI database. Taxonomic classification was performed with the default “h + p + v + c” database and standard parameters (Centrifuge “-k 1”) on fastq reads using centrifuge pipeline [18]. Bacterial abundance profiles were retrieved from the Centrifuge reports. Porechop v0.2.3 (https://github.com/rrwick/Porechop) was used to further assess barcodes in the reads. Relative taxonomic abundances of reads from Centrifuge data were visualized using Krona hierarchical data browser [19] produced by the Krona tools scripts (http://sourceforge.net/projects/krona/). The results of centrifuge pipeline were also analysed and visualized with the Pavian tool [20]. Relative abundances were also visualized at all taxonomic levels by stacked bar plots using the *ggplot2* R package (http://cran.r-project.org/package=ggplot2), and by heatmaps using m*atplotlib* and *seaborn* Python libraries. Alpha diversity (Shannon diversity) indices were calculated by boxplots using *ggplot2* and *beeswarm* R libraries (http://cran.r-project.org/package=beeswarm). The 3-dimensional principal coordinates analyses (PCoA) were visualized using m*atplotlib* and s*eaborn* Python libraries.

### 3.6 Predictive metabolic inference using 16S rRNA data

PAPRICA software (PAthway PRediction by phylogenetIC placement) [21] was used to predict metabolomic functions/pathways of gut microbial communities based on taxonomical information. PAPRICA provides metabolic inference, based on 16S rRNA gene libraries and predicts the function of bacteria involved in metabolic pathways [[22], [23], [24], [25], [26], [27]].

### 3.7 Statistical analysis

Data was analysed and visualized using the R software version 4.3.2 (http://www.r-project.org). A p-value <0.05 was considered statistically significant. Clinical and demographic data were presented either as mean or median values with range for quantitative variables and absolute numbers in percent for categorical variables. Categorical data were compared using chi-square or Fisher's exact tests, while continuous variables were compared using t-tests or Mann Whitney U-tests as appropriate.

### 3.8 Role of funders

The funders were not involved in study design, data collection, analysis, interpretation or writing.

## 4 Results

### 4.1 Baseline characteristics

Of the 48 samples from children included in this study, 12 were with diarrhoea due to RVA infection (mean 16.5 ± 15 months), 12 were with diarrhoea and without RVA infection (mean 18.5 ± 17 months), 12 were with no diarrhoea, but with RVA infection (mean 29 ± 15 months), and 12 were from healthy controls with no diarrhoea and no RVA infection (mean 31 ± 15 months). The demographic and clinical characteristics of 48 enrolled children are summarised in Table 1. While diarrhoea lasted four complete days, the mean diarrhoea frequency per day was 3.8 and 3.3 in RVA-positive and negative children, respectively. The mean age in the diarrhoea group was significantly lower than in the non-diarrhoea groups (p-value 0.007, 2-tailed *t*-test).

**Table 1:** Demographic and clinical characteristics of the enrolled Gabonese children.

	Diarrhoea		Non-diarrhoea	
	RVA positive (n = 12)	RVA negative (n = 12)	RVA positive (n = 12)	RVA negative (n = 12)
Mean age in months	16.5	18.5	29	31
Median age (IQR) in months	13.5 (2–59)	14.0 (5–59)	30 (12–48)	36 (2–48)
Female/Male	6/6	9/3	3/9	7/5
Rural/Urban resident	5/7	12/0	10/2	7/5
Mean frequency of diarrhoea/day	3.8	3.3	NA	NA
Mean duration of diarrhoea (days)	4	4	NA	NA
Bloody diarrhoea	3	7	NA	NA
Mild dehydration	9	7	NA	NA
Moderate dehydration	0	3	0	NA
Vomiting	6	8	0	NA
Presenting with fever	6	5	0	NA
Presenting with anaemia	9	6	0	NA

RVA -rotavirus A; NA – not applicable.

### 4.2 Gut microbiota diversity

**Fig. 2:**
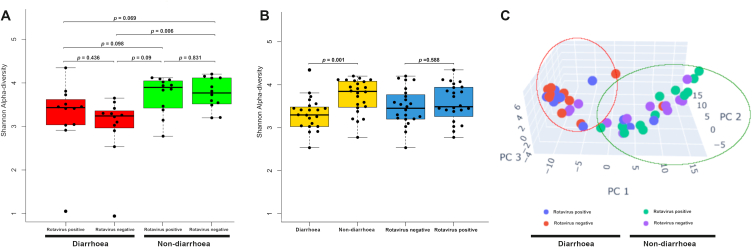
Alpha and beta diversity among the study groups. **(A)** Shannon alpha diversity **(B)** Shannon alpha diversity of study groups. **(C)** Principal Coordinates Analysis (PCoA) of beta-diversity.

To understand the gut microbiota of 48 study subjects, the alpha diversities were analysed using the Shannon diversity index (Fig. 2A and B). The alpha diversity of the diarrhoea groups (diarrhoea with RVA and diarrhoea without RVA) was lower than that of the non-diarrhoea groups (Fig. 2A), suggesting that the diarrhoea groups had low bacterial abundance and diversity. However, pairwise *t*-test between the four groups showed a significant difference in alpha diversity between the group with diarrhoeal disease without RVA and the healthy controls (p = 0.006, 2-tailed *t*-test) (Fig. 2A). Subsequently, samples were regrouped into diarrhoeal, non-diarrhoeal, rotavirus A-positive, and rotavirus A-negative groups and were analysed for alpha diversity indices (Fig. 2B). We found significantly low diversity (p = 0.001, 2-tailed *t*-test) between diarrhoeal and non-diarrhoeal group.

Next, beta-diversity analysis of bacterial communities among the study groups was performed using Principal Coordinates Analysis (PCoA). The PCoA plot showed a distinct clustering pattern between diarrhoeal and non-diarrhoeal samples (Fig. 2C). The diarrhoea groups clustered, while the non-diarrhoea groups were heterogeneously distributed, suggesting that the microbiota in the non-diarrhoea samples was very diverse, while the diarrhoea samples were less diverse.

### 4.3 Gut microbiota community composition

**Fig. 3:**
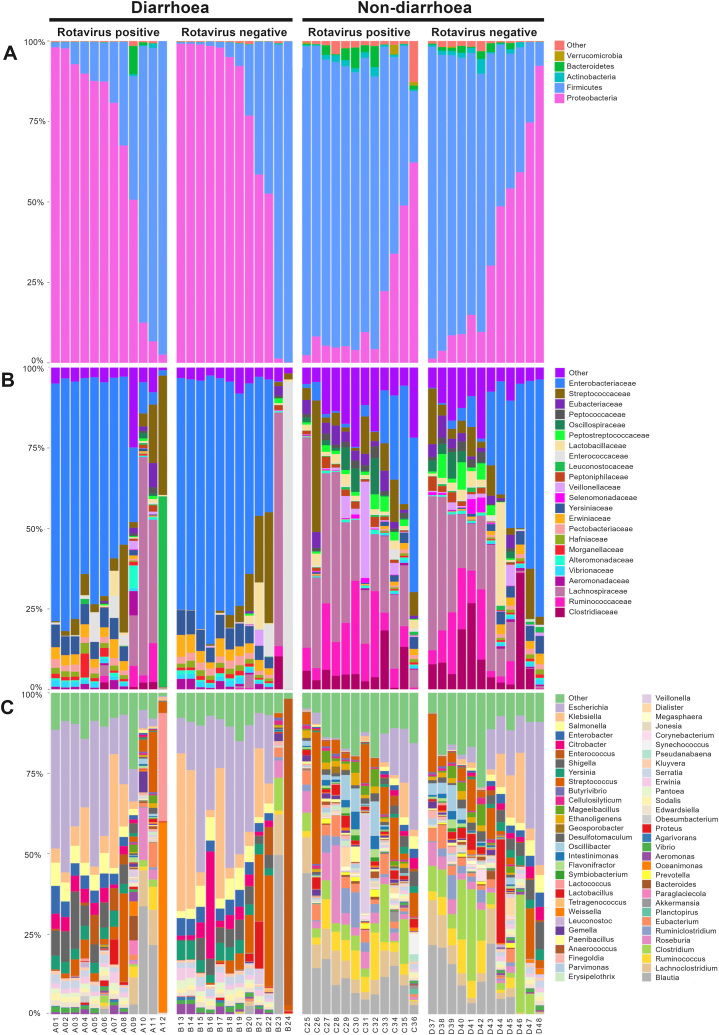
Gut microbial composition of the investigated study groups. Each stacked bar represents the mean relative abundance of bacterial genome in each group. **(A)** Taxonomic assignment by phylum. **(B)** Taxonomic assignment by family **(C)** Taxonomic assignment by genus.

**Fig. 4:**
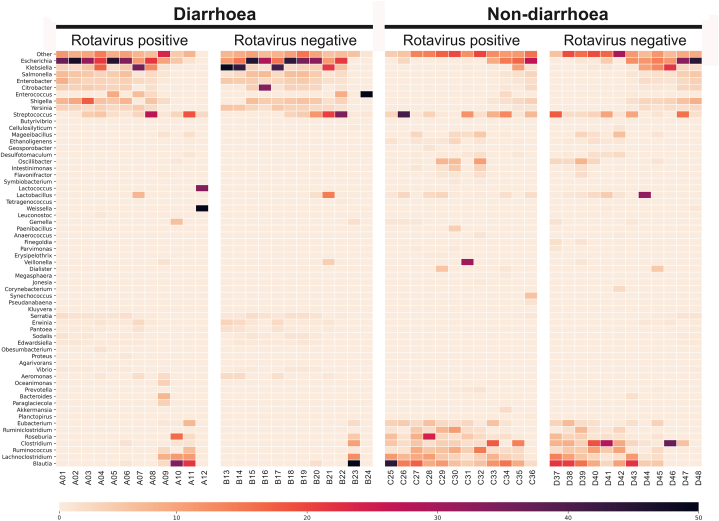
Heatmap of the frequency of the 30 most common bacterial species associated with diarrhoea. The heatmap shows the relative percentage of bacterial species within each group, as shown in a grid for each sample. The darker the colour, the higher the abundance.

Taxonomic comparisons were made among the four groups at the phylum, family, and genus levels. In general, at phylum level the most predominant bacteria found among all groups were Firmicutes (50%), and Proteobacteria (47%) (Fig. 3A). Members of the Proteobacteria (68%) were most abundant in the diarrhoeal groups, while *Firmicutes* (69%) were the predominant in the non-diarrhoeal groups. Proteobacteria (53%) were more present in children not infected with RVA, while the proportion of Firmicutes (55%) was greater in children infected with RVA. In addition to the phylum, the bacterial families were also analysed. The percentages of the occurrence of the 23 predominant families, along with other communities present in lesser proportions, were observed (Fig. 3B). At the family level, *Enterobacteriaceae* (49%) were most abundant, along with enrichment of *Yessiniaceae* (5%) and *Erwiniaceae* (3%) in the diarrhoea groups, while *Lachnospiraceae* (24%) were most abundant, along with enrichment of *Enterobacteriaceae* (17%), *Ruminococcacceae* (10%), and *Clostridiaceae* (8%) in the non-diarrhoea groups (Fig. 3B). In addition, results showed *Enterobacteriaceae, Yessiniaceae,* and *Erwiniaceae,* which were most common in the diarrhoea group were less common in RVA-infected children. The predominant families in the non-diarrhoea group were *Lachnospiraceae, Ruminococcacceae* and *Clostridiaceae* and were most common in RVA-infected children. At the genus/species level, we observed *Escherichia* (21%), *Klebsiella* (10%) and *Salmonella* (4%) being the most abundant in the diarrhoeal groups, while the genera *Blautia* (11%), *Clostridium* (8%), *Escherichia* (8%), *Lachnoclostridium* (6%) and *Ruminococcus* (5%) were abundant in the non-diarrhoea groups (Fig. 3C). All the predominant genera in the diarrhoea groups were also found in abundance in children not infected with RVA. Conversely, those predominant in the non-diarrhoea groups were found in significant proportions in children infected with RVA. At the genus level (Supplementary Table 1), a total of 11 major genera showed a significant difference between the RVA-positive group with diarrhoea and the RVA-positive group without diarrhoea, while a total of 16 major genera showed a significant difference between the RVA-negative group with diarrhoea and the RVA-positive group without diarrhoea (Supplementary Table 2). To understand which bacterial species causing diarrhoea are enriched in the study groups, we chose 30 most common diarrhoea-causing bacteria from literature [[28], [29], [30]] and examined their abundance (Fig. 4). The heat map showed the three most common bacterial species, namely *E. coli, K. pneumoniae* and S. *enterica*, in the diarrhoea groups.

### 4.4 Metabolites composition in diarrhoeal and non-diarrhoeal children

**Fig. 5:**
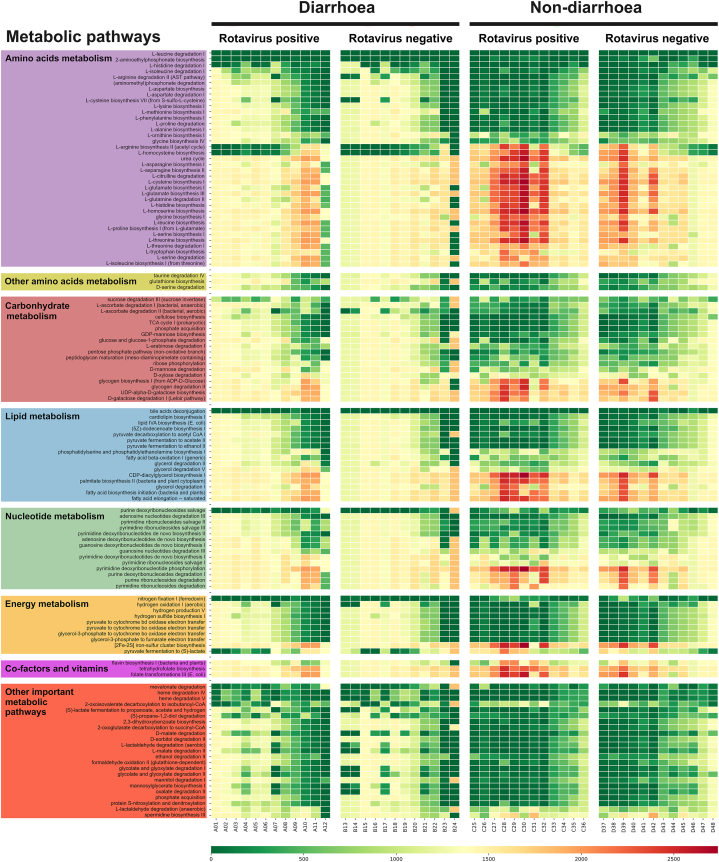
Relative abundance heatmap of metagenome function as predicted by analysis with PAPRICA (PAthway PRediction by phylogenetIC placement).

The microbiota produces metabolites and these metabolites influence the physiological functions of the host through their metabolic activity [31]. The analysis showed that 960 metabolites were quantitatively altered among the groups, of which 124 metabolites were involved in amino acid, carbohydrate, lipid, nucleotide, and vitamin metabolism (Fig. 5). The diarrhoeal groups showed the lowest spectrum than the non-diarrhoeal groups. The ANNOVA analysis between groups revealed significant differences between the diarrhoeal and non-diarrhoeal with RVA infection. No significant differences were observed either for the diarrhoeal or non-diarrhoeal groups without RVA infection. Significant differences were found for 32 metabolites from the amino acid domain, 15 from the carbohydrate domain, eight from the lipid domain and nine from the energy-specific metabolic pathways (Supplementary Table 3).

## 5 Discussion

The gut microbiome is a complex ecosystem influenced by many factors and characterised by high bacterial diversity and gene richness. Perturbations caused by enteric infections such as RVA can affect the microbial diversity and abundance in the gut, and cause dysbiosis of the gut microbiome [32,33]. Few studies have examined the microbiota of children with rotavirus, and most of these have focused on examining the effects of RotaTeq® vaccine against RVA on the gut microbiota [14,[34], [35], [36], [37]]. Our study focuses on the RVA vaccine-naïve population and provides the first insights into the altered microbiota in RVA-related enteric infections, particularly in children under five years of age. Notably, significant differences in gut microbiome composition were observed only in children with diarrhoea, while no significant differences in gut microbiota composition were observed in the context of RVA-infected children with or without diarrhoea.

Alpha diversity, indicating species diversity or species richness, showed that healthy children had high microbial diversity compared to children with acute diarrhoea. Children with acute diarrhoea without RVA infection had low microbial diversity compared to healthy children, indicating dysbiosis of the gut microbiota. This phenomenon has been well described and shows that repeated flushing during acute diarrhoeal illness severely erodes the microbiota and that higher faecal water content reduces alpha diversity [38]. Also, the findings are consistent with the observation of Ma et al. reporting that rotavirus can alter the human gut microbiome by increasing opportunistic pathogens [12].

The gut microbiota of children is largely colonized by bacteria of the phyla Bacteroidetes, Firmicutes, and Proteobacteria [39]. In the analysis of beta diversity, which indicates the species diversity between two bacterial communities, a high bacterial heterogeneity in children without diarrhoea compared to children with diarrhoea was observed. Proteobacteria (68%) were most abundant in the diarrhoeal groups, while Firmicutes (69%) were the predominant in the non-diarrhoeal groups. Another interesting observation is that Proteobacteria (53%) were more present in children not infected with RVA, while the proportion of Firmicutes (55%) was greater in children infected with RVA. As reported by Shin et al. [40], the high abundance of Proteobacteria found in this study may be a sign of an imbalanced microbiome due to an infection, as observed in the group of children with diarrhoea, some of whom were infected with RVA. This is consistent with the study by Sohail et al. which showed that RVA infections are positively associated with an increased proportion of Proteobacteria [14]. Engevik et al. have also shown that there are shifts at the phylum level in the ileum on the first day after rotavirus infection, i.e. the presence of Proteobacteria, Bacteroidetes and Verricomicrobia increases, while the presence of Firmicutes decreases compared to controls [41]. In our case, this would clearly explain the variation of certain taxa in relation to the RVA status of the children. For example, the proportion of Firmicutes was 55% in RVA-infected children, while it was 45% in uninfected children.

*Escherichia* (21%), *Klebsiella* (10%) and *Salmonella* (4%) being the most abundant in the diarrhoeal groups, while the genera *Blautia* (11%), *Clostridium* (8%), *Escherichia* (8%), *Lachnoclostridium* (6%) and *Ruminococcus* (5%) were abundant in the non-diarrhoea group. The predominance of *E. coli*, *Salmonella* and *Klebsiella* pathogens found in infants with diarrhoea in this study is consistent with what has been reported in previous studies [[42], [43], [44]], in which *E. coli* and *Klebsiella* were observed, and was also reported by Lil et al. in infants with rotavirus-induced diarrhoea [45].

Microbial metabolic pathways and compounds facilitate digestion and absorption of nutrients from food while promoting maturation and proper immune system function [31]. As the taxonomic richness and diversity in the diarrhoea groups were low compared to the healthy controls, we sought to understand the metabolite composition in children with and without diarrhoea. Of the 960 metabolites were quantitatively altered, significant differences due to diarrhoeal disease were found for 32 metabolites from the amino acid domain, 15 from the carbohydrate domain, eight from the lipid domain and nine from the energy-specific metabolic pathways. This could be explained by an alteration of the metabolome due to diarrhoea-induced microbiota dysbiosis and is in line with recent studies showing that metabolic changes and disturbances of the gut microbiota run in parallel during disease progression [46,47].

The median age in the RVA-positive diarrhoea group and the RVA-negative diarrhoea group was 13.5 and 14 months, respectively, while in the RVA-positive non-diarrhoea group and the RVA-negative non-diarrhoea group it was 30 and 36 months, respectively. A first limitation of our study is the significant age differences in months were observed between the groups with and without diarrhoea, which could partially influence on the composition of the microbiota. Secondly, lack of data on nutritional status, antibiotic treatment, probiotic administration, and breastfeeding, which might have a significant impact on the microbiome. Another limitation is that only diarrhoea caused by RVA was investigated in this study, so that other causative protozoa such as *Cryptosporidium* sp., *Giardia lamblia*, *Entamoeba histolytica* and enteric viruses such as norovirus, sapovirus, astrovirus, if present, could have caused microbial dysbiosis in the gut.

In summary, diarrhoea per se affects the diversity and richness of the gut microbiota. More importantly, there is a clear association between RVA infection and the relative richness of the gut flora in vaccine-naive Gabonese children. Taken together, diarrhoea and RVA infection are two independent factors altering the gut microbiome. Elucidating the impact of the microbiota on immune homeostasis and its influence on enteric viral infections is another challenge where large parts of sub-Saharan Africa have not adopted rotavirus vaccination.

## Contributors

TPV and SK conceptualized and designed the study. AAA contributed to the study design. TPV and SRP supervised the study procedures. AAA and PGM recruited the patients. SK, GPM, LTLK performed the experimental work. PGM, SK and SRP performed the data analysis. SK, GPM, SRP, TPV wrote the first draft. TPV, SN and PGK revised the manuscript. All authors have read and approved to the published version of the manuscript.

## Data availability statement

All data are available within the manuscript. Additional microbiome data from this study can be retrieved from European Nucleotide Archives (ENA) and are available under accession numbers ERR11758216 to ERR11758263.

## CRediT authorship contribution statement

**Gédéon Prince Manouana:** Writing – review & editing, Resources, Methodology, Investigation, Data curation. **Salih Kuk:** Writing – original draft, Visualization, Methodology, Investigation, Formal analysis, Data curation, Conceptualization. **Le Thi Kieu Linh:** Methodology, Investigation. **Srinivas Reddy Pallerla:** Visualization, Validation, Supervision, Methodology, Investigation, Formal analysis, Data curation. **Sandra Niendorf:** Writing – review & editing. **Peter G. Kremsner:** Writing – review & editing. **Ayola Akim Adegnika:** Supervision, Resources, Project administration, Funding acquisition. **Thirumalaisamy P. Velavan:** Writing – review & editing, Writing – original draft, Validation, Supervision, Resources, Project administration, Methodology, Funding acquisition, Conceptualization.

## Declaration of competing interest

The authors declare that they have no known competing financial interests or personal relationships that could have appeared to influence the work reported in this paper.
